# Intrinsic Functional Connectivity Network in Children with Dyslexia: An Extension Study on Novel Cognitive–Motor Training

**DOI:** 10.3390/brainsci16010055

**Published:** 2025-12-30

**Authors:** Mehdi Ramezani, Angela J. Fawcett

**Affiliations:** 1 Independent Researcher, Tehran 1865987169, Iran; 2 Department of Psychology, Swansea University, Swansea SA1 8EN, UK

**Keywords:** intrinsic connectivity, functional connectivity, brain network, fMRI, cognitive–motor, training program, dyslexia

## Abstract

**Objectives**: Innovative, evidence-based interventions for developmental dyslexia (DD) are necessary. While traditional methods remain valuable, newer approaches, such as cognitive–motor training, show the potential to improve literacy skills for those with DD. Verbal Working Memory–Balance (VWM-B) is a novel cognitive–motor training program that has demonstrated positive effects on reading, cognitive functions, and motor skills in children with DD. This extension study explored the neural mechanisms of VWM-B through voxel-to-voxel intrinsic functional connectivity (FC) analysis in children with DD. **Methods:** Resting-state fMRI data from 16 participants were collected in a quasi-double-blind randomized clinical trial with control and experimental groups, pre- and post-intervention measurements, and 15 training sessions over 5 weeks. **Results:** The mixed ANOVA interaction was significant for the right and left postcentral gyrus, bilateral precuneus, left superior frontal gyrus, and left posterior division of the supramarginal and angular gyri. Decreased FC in the postcentral gyri indicates reduced motor task engagement due to automation following VWM-B training. Conversely, increased FC in the bilateral precuneus, left superior frontal gyrus, and left posterior divisions of the supramarginal and angular gyri suggests a shift of cognitive resources from motor tasks to the cognitive functions associated with VWM-B. **Conclusions:** In conclusion, the study highlights that cognitive–motor dual-task training is more effective than single-task cognitive training for improving cognitive and motor functions in children with DD, emphasizing the importance of postural control and automaticity in dyslexia. The trial for this study was registered on 8 February 2018 with the Iranian Registry of Clinical Trials (IRCT20171219037953N1).

## 1. Introduction

Developmental dyslexia (DD) has been defined by various hypotheses over the past four decades, yet there are common viewpoints for its definition. These individuals, despite having adequate intellectual abilities, struggle to read accurately and fluently and comprehend text [1,2]. The prevalence of DD is about 7% in primary school children but can range from 1.3% to 17.2% [3,4]. Untreated DD can significantly affect children’s abilities in education, careers, communication, and mental health [5]. It may lead to school refusal, as poor academic performance makes school an unpleasant experience [6]. Children with DD are less likely to complete school or pursue higher education [7]. Therefore, introducing early, evidence-based interventions is important for improving the quality of life for individuals with dyslexia and reducing its long-term effects [8,9].

Researchers have examined various interventions to improve phonological awareness, phoneme-to-grapheme conversion, working memory, vestibular function, visuomotor and visuospatial processing, and postural control in dyslexia [10,11,12,13,14,15,16,17,18,19,20]. Traditional methods, like phonics instruction and individualized education plans, remain important, but the rise of innovative therapeutic modalities highlights the need for updated treatment strategies [21]. Given that the traditional interventions have typically been unimodal and single-task, future strategies should be multimodal, multisensory, individualized, adaptive, and based on solid evidence [9,22,23]. Newer approaches, including computer-assisted and cognitive–motor therapies, have demonstrated the potential to improve literacy skills for individuals with DD [24].

Cognitive–motor training has demonstrated positive effects on cognitive abilities, balance, and executive functions in various populations [25,26]. However, there is insufficient evidence to determine the most effective training methodology, prompting a call for the development of novel cognitive–motor interventions [27]. It is recommended that these types of interventions incorporate adequate difficulty, intensity, duration, task specificity, and task prioritization [28]. Verbal Working Memory–Balance (VWM-B) is a newly developed dual-task cognitive–motor training program with a unique structure [24]. This program possesses different difficulty levels, intensities, durations, task specificities, and priorities [24]. Recent clinical trials have shown its positive effects on cognitive functions (attention, working memory, executive functions) and motor skills (balance and postural control) in children with DD [2,24,29]. VWM-B has significantly enhanced reading skills and text comprehension in those children [2,24,29]. In a randomized clinical trial, alterations in resting-state functional connectivity (FC) of the left fusiform gyrus were reported in children with DD following VWM-B training, using ROI-to-ROI mixed ANOVA analysis [2]. The results have indicated increased resting-state FC of the left fusiform gyrus with the cerebellar (Crus II regions) and cerebral regions (left anterior temporal fusiform cortex and the left middle frontal gyrus) [2]. The ROI-to-ROI mixed ANOVA analysis in that study has highlighted the crucial role of the left fusiform gyrus in reading ability and has supported the presence of cerebellar deficits in dyslexia [2].

The ROI-to-ROI method employs a seed-to-seed approach, selecting predefined ROIs based on prior knowledge and assessing FC through the correlation of time series data between these regions [2]. This technique simplifies analysis, supports hypothesis testing, and enhances interpretability [30,31]. However, it may miss significant connectivity patterns outside the predefined ROIs, potentially overlooking critical interactions among brain regions [31,32]. In contrast, the voxel-to-voxel approach analyzes FC at a finer spatial resolution, assessing the correlation of time series data between all voxels in the brain without prior segmentation into ROIs [31,32]. Therefore, this method has a holistic view, which captures the entire brain’s connectivity patterns, allowing for the discovery of novel connections that may not be evident in ROI-based analyses [31,32]. In other words, it is a data-driven method and does not rely on predefined regions, which can help in identifying unexpected patterns or networks that emerge from the data itself [31,32]. Given that there is only one neuroimaging study on the VWM-B program’s underlying neural mechanism [2], voxel-to-voxel intrinsic FC was used as a principled exploratory step to identify connectivity alterations without imposing strong a priori assumptions. In future studies, therefore, the outcomes of the present study can consequently be examined using seed-based, network-level, or other neuroimaging analysis techniques, enhancing our understanding of the neural mechanisms underlying the VWM-B program.

This research is an extension study built on the original work by Ramezani et al. (2023) [2], focusing on the investigation of the neural mechanisms underlying the VWM-B program in children with DD. We conducted a new analysis on the resting-state fMRI data from the original study, employing voxel-to-voxel intrinsic FC analysis to identify a VWM-B-induced network in children with DD. Unlike the ROI-to-ROI analysis in the original study, the new approach estimates an intrinsic FC network between each voxel and all other voxels in the brain.

## 2. Methods

### 2.1. Methodology Overview of the Original Study

**Figure 1:**
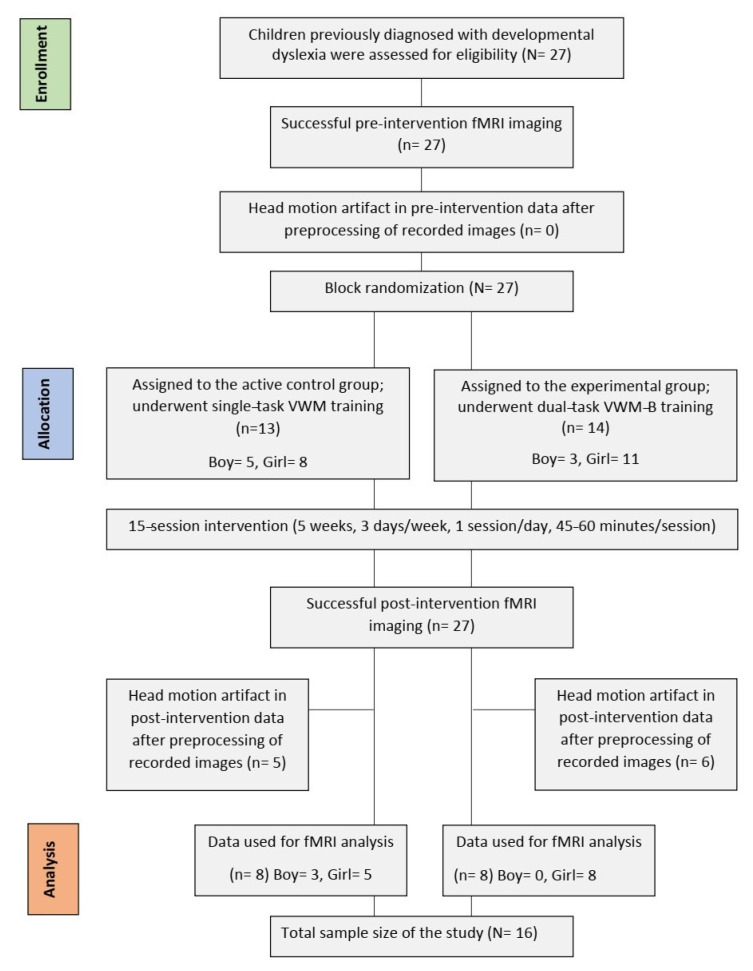
The participant recruitment procedure for the study.

The original study was a quasi-double-blind randomized clinical trial that included a between-subjects factor (‘group’: control and experimental) and a within-subjects factor (‘time’: pre and post-intervention measurements) [2]. The trial for this study was registered on 8 February 2018 with the Iranian Registry of Clinical Trials (IRCT20171219037953N1). It aimed to explore the neural mechanisms of the VWM-B program (experimental group) by comparing its effectiveness with the VWM program (active control group), a traditional single-task training program for children with DD [2]. Behavioral data from 27 participants aged 7 to 9 years were analyzed; these children were randomly assigned to groups and underwent baseline testing, with 15 training sessions over 5 weeks (3 days per week, 45 to 60 min per day), and a post-intervention assessment [2]. For fMRI data analysis, only data from 16 subjects were included due to the absence of head motion artifacts [2]. Eleven participants were excluded for exceeding a median framewise displacement of 3 mm [mean (SD) = 7.29 (5.41)], resulting in 8 children in each group [2]. There was no significant difference in framewise displacement between the groups (*p* > 0.05) [2]. Notably, the fMRI data from these groups were employed in the present study. A post hoc power analysis indicated a power of 79% for the total sample size of 16, assuming a significance level of 0.05 [2]. Additionally, the groups were matched for potential confounding variables such as age, gender, full IQ, and attention (*p* > 0.05). See Figure 1 for details on the participant recruiting procedure in accordance with CONSORT guidelines.

Alongside the fMRI data, the original study employed questionnaires and a force plate tool to gather center of pressure (CoP) data [2]. The questionnaires included the Wechsler intelligence scale for children, fourth edition, for IQ estimation, the child symptoms inventory for evaluating attention, NEMA reading subtests for assessing reading skills, the forward digit span for verbal working memory capacity, and the Stroop color word test for estimating other cognitive functions [2].

Inclusion criteria included normal vision and hearing, normal attention and normal IQ scores, right-handedness, average socio-economic status, and being a native Persian language speaker [2]. Exclusion criteria included a history of neurological or psychiatric disorders, taking drugs that affect the central nervous system, and failing to complete a minimum of 75% of the training sessions [2]. For double-blinding, participants/parents and data evaluators/analyzers were unaware of the assigned interventions in the groups [2].

For a detailed explanation of the training programs (VWM-B and VWM) used in the study, refer to Ramezani et al., 2021 [24]. The VWM program utilized a 19-inch touchscreen monitor and a speaker for word recitation, while the child sat comfortably with their arms on a table [24]. In contrast, the VWM-B program employed a portable robotic device featuring a tiltable platform that could adjust between 0 and 20 degrees. This platform included a force plate to track the participant’s CoP with 100 Hz accuracy (±0.4 mm), a speaker for word recitation, and a 19-inch touchscreen interface for software control [24]. For safety, each participant’s limit of stability (LoS) was calibrated before they began training on the robot [24]. The VWM and VWM-B programs encompass the encoding, maintenance, and retrieval processes of working memory and were each operated using different computer software [24]. In both programs, during the encoding step of working memory, the target—a word, a series of words, or a statement—was displayed inside the target box on the monitor for ten seconds [24]. Simultaneously, a pre-recorded voice on the computer recited the target [24]. In the maintenance step of working memory in the VWM program, the target box’s content was broken down into its components (sentence into words or word into letters) and displayed on the monitor in component boxes for ten seconds [24]. In the VWM-B program’s maintenance step, a CoP marker appeared on the screen alongside the component boxes to indicate the subject’s CoP position [24]. A novel addition to the VWM-B program was the inclusion of two phases in the maintenance step: passive and active states of balance [24]. In the passive state, the motorized platform tilted, causing the CoP marker to move toward the component boxes, creating a passive exercise [24]. In the active state, the participant actively moved their CoP towards the component boxes, with the platform remaining stationary without tilting [24]. In the final step of the working memory, the retrieval step, a doubled number of boxes, containing both practiced components and new ones, appeared on the monitor for ten seconds [24]. Participants in the control group (VWM) selected and touched answer boxes on the monitor. On the other hand, participants in the experimental group (VWM-B) chose response options by utilizing CoP movements [24].

### 2.2. MRI Acquisition and Parameters

The scanning was performed on a SIEMENS MAGNETOM Prisma 3.0T MRI scanner with a 20-channel head coil at the National Brain Mapping Laboratory (NBML), Tehran, Iran [2]. The participants were instructed to lie still with their eyes closed during scanning, with a resting state without falling asleep [2]. To make participants comfortable in the scanner, they were familiarized with the MRI process, the scanner room was decorated to look like a sea with the scanner as a submarine, and videos also showed other children in the scanner [2]. During scanning, foam cushions and a headband were used to limit head movement while data was acquired [2]. Employing a T2*-weighted echo-planar imaging sequence, resting-state fMRI images were obtained with an echo time of 26 ms, a repetition time of 2000 ms, a flip angle of 60 degrees, a 234 × 234 mm^2^ field of view, and 33 transverse slices covering the whole brain at 3 mm thickness, with a 78 × 78 matrix size [2]. Each functional scan lasted 6 min and 46 s. High-resolution T1-weighted structural whole-brain images were also collected using a 3D magnetization prepared rapid acquisition gradient echo pulse sequence with a repetition time of 1850 ms, an echo time of 3.53 ms, a flip angle of 7 degrees, 176 slices at 1 mm thickness, and a 244 × 244 mm^2^ field of view [2]. Each anatomical scan lasted 4 min and 19 s [2]. These structural scans were utilized to co-register and normalize the resting-state fMRI data [2].

### 2.3. Image Preprocessing and Analyses

The CONN (v.17.f) toolbox (https://www.nitrc.org/frs/?group_id=279 (accessed on 19 December 2018) was used to preprocess and analyze the voxel-to-voxel intrinsic FC of the preprocessed images [2]. Intrinsic FC demonstrates moderate to high reliability in studies with both within- and between-subject designs [33]. The reconstructed fMRI data underwent several preprocessing steps: (1) slice timing correction, (2) motion correction through realignment and unwarping using the first image of the session, (3) segmentation into gray matter, white matter, and cerebrospinal fluid, (4) normalization to the Montreal Neurological Institute (MNI) space for subjects aged five and above [34], and (5) spatial smoothing with an 8 mm full width at half-maximum (FWHW) Gaussian kernel [2]. A 3D affine transformation was applied for aligning volumes, accounting for three translational and three rotational motion parameters [2]. Since head motion artifacts are a confounding factor for intrinsic FC analysis of MRI images, especially in pediatric populations, subjects with a median voxel displacement of >3 mm were excluded from the post-processing pipeline [2,35]. Outlier volumes were identified using the ART-based scrubbing procedure implemented in the CONN toolbox and were included as nuisance regressors during the denoising step. Prior to intrinsic FC analysis, additional temporal processing was conducted using an aCompCor strategy along with a band-pass filter of 0.008–0.09 Hz [36,37,38]. This involved averaging signals from white matter, cerebrospinal fluid, and whole-brain data, as well as parameters from the six head motion directions and their first-order derivatives to eliminate linear trends through multiple regression analysis [2,33].

The root mean square of the correlation coefficients between an individual voxel and all voxels in the brain defines intrinsic FC [37]. Voxel-to-voxel intrinsic FC was computed using data-driven functional connectivity multivariate pattern analysis (FC-MVPA) framework implemented in the CONN toolbox. For each voxel, whole-brain connectivity profiles were generated using principal component analysis following the default CONN implementation. First- and second-level analyses were performed using standard methods from the CONN toolbox [37]. The first-level analysis examined the similarity of global FC patterns between each voxel and its neighbors, rather than focusing on a specific seed region (ROI-to-ROI analysis in the original paper) [38]. The first-level FC maps were then used in a voxel-to-voxel whole-brain second-level analysis employing a between-within ANOVA, with a between-subjects contrast of [1 −1] and a between-condition contrast of [−1 1]. Connectivity values were Fisher’s z-transformed prior to group-level mixed-design ANOVA analyses. Fisher’s z-transformed intrinsic FC maps from all participants were analyzed using a general linear model (GLM) to assess time and group differences in intrinsic FC [2]. This second-level analysis compared voxel-to-voxel connectivity maps across participants and conditions. Consistent with the voxel-to-voxel FC-MVPA approach, no a priori seed interest regions or predefined network labels were applied in order to preserve the data-driven nature of the analysis. An FDR-corrected *p*-value of <0.05 was determined as the threshold for statistical significance [2].

## 3. Results

The study involved 16 children with DD, with a mean ± SD age of 8 ± 1.15 years. The control group comprised eight children with DD (mean ± SD age: 8 ± 1.24 years), and the experimental group also included eight children with DD (mean ± SD age: 8 ± 1.13 years). Both groups were matched for potential confounders, including age, IQ, and attention level (*p* > 0.05). Before evaluating the group differences, the normality of the variables was assessed by the Shapiro–Wilk test. Despite the control group having three male participants compared to none in the experimental group, no statistically significant differences between the groups were found using the chi-square test (*p* = 0.055). For more information, refer to Table 1 for demographic characteristics.

After training with the programs, mixed ANOVA was used to estimate the voxel-to-voxel intrinsic FC network. The results indicated that the VWM-B program, in comparison to the VWM program, modifies the activation of certain brain regions that were identified as an intrinsic FC network for VWM-B. Table 2 lists these regions in descending order of effect sizes (Beta). The interaction was found to be significant for the right postcentral gyrus [Beta (T) = −2.13 (−5.55), *p*-FDR = 0.000], left postcentral gyrus [Beta (T) = −1.91 (−5.46), *p*-FDR = 0.000], left/right precuneus [Beta (T) = 1.54 (5.55), *p*-FDR = 0.000], left superior frontal gyrus [Beta (T) = 1.43 (8.06), *p*-FDR = 0.000], and left posterior division of supramarginal gyrus/left angular gyrus [Beta (T) = 1.27 (7.50), *p*-FDR = 0.000]. After VWM-B training, intrinsic FC decreased in the right and left postcentral gyri but increased in the left and right precuneus, left superior frontal gyrus, and the left posterior division of the supramarginal and angular gyri.

**Figure 2:**
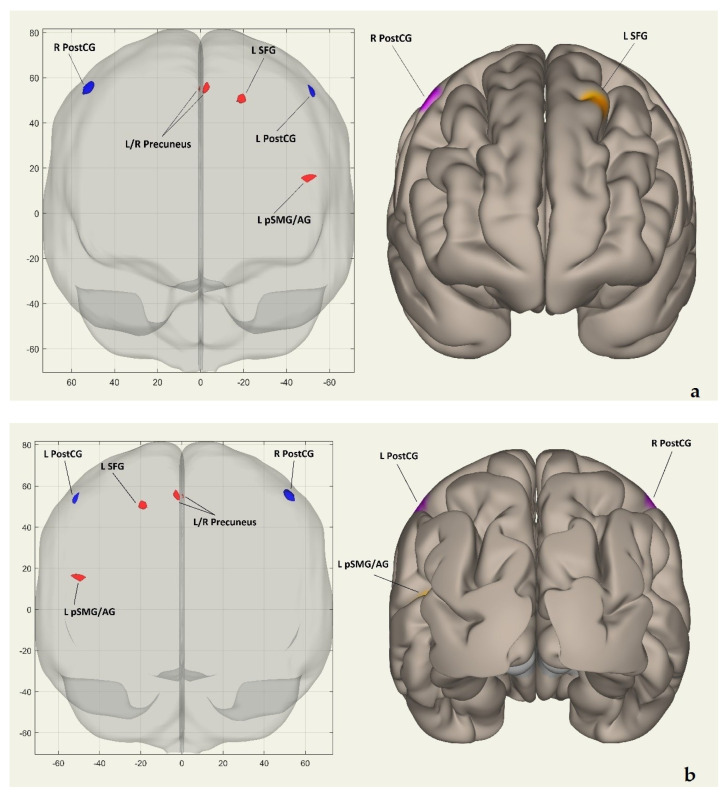

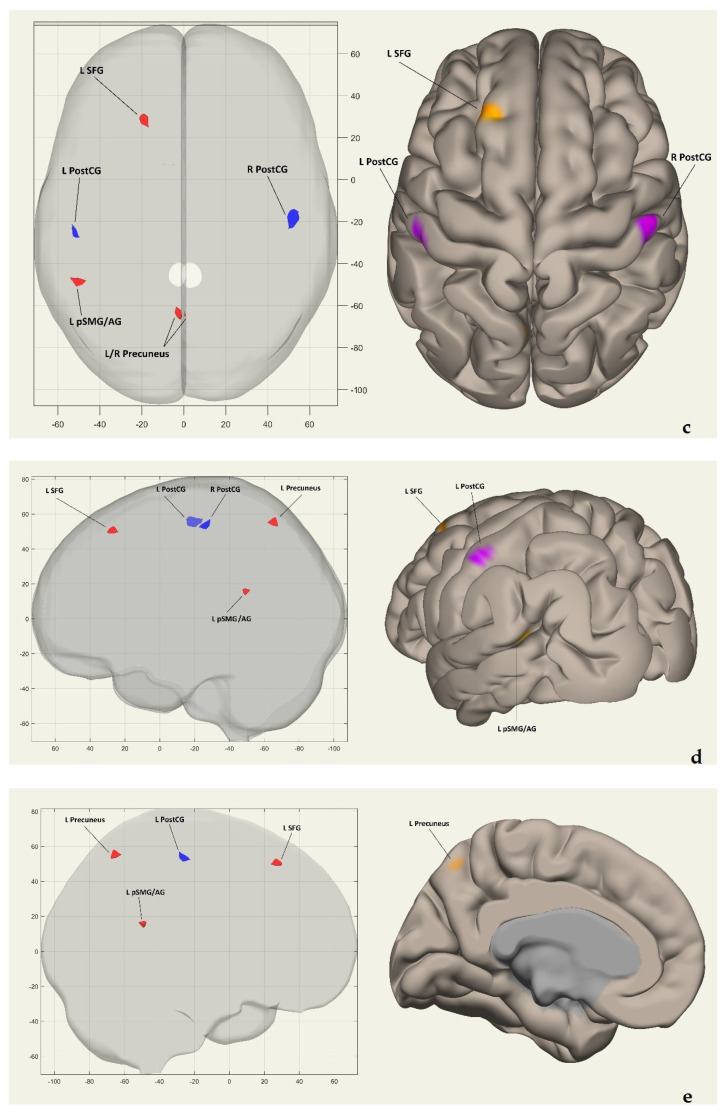

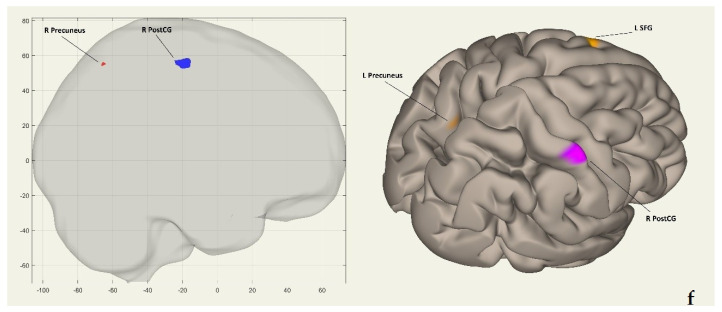
(**a**) Coordinates (x, y, z) of clusters in the voxel-to-voxel intrinsic functional connectivity network shown in coronal and frontal views. (**b**) The coordinates (x, y, z) of clusters in the voxel-to-voxel intrinsic functional connectivity network are shown from coronal and posterior views. (**c**) Displays the coordinates (x, y, z) of clusters in the voxel-to-voxel intrinsic functional connectivity network from horizontal and superior views. (**d**) Coordinates (x, y, z) of clusters in the intrinsic functional connectivity network on the sagittal plane and left hemisphere views are displayed. (**e**) Coordinates (x, y, z) of clusters in the voxel-to-voxel intrinsic functional connectivity network displayed in sagittal and left hemisphere medial views. (**f**) Coordinates (x, y, z) of clusters in the voxel-to-voxel intrinsic functional connectivity network, shown from sagittal and right hemisphere lateral views. In all Figure 2a–f, red/orange clusters indicate positively activated regions, while blue/purple clusters represent negatively activated regions. Abbreviations: R PostCG—right postcentral gyrus; L PostCG—left postcentral gyrus; L SFG—left superior frontal gyrus; L pSMG/AG—left posterior supramarginal/angular gyrus; L/R precuneus—left/right precuneus; L precuneus—left precuneus; R precuneus—right precuneus.

Figure 2a shows the clusters’ coordinates (x, y, z) of the intrinsic FC network in coronal and frontal views. Figure 2b displays the same coordinates in coronal and posterior views. Figure 2c presents the coordinates on the horizontal plane in superior views. Figure 2d exhibits them on the sagittal plane in left hemisphere lateral views, while Figure 2e demonstrates the coordinates in left hemisphere medial views. Finally, Figure 2f showcases the clusters on the sagittal plane in right hemisphere lateral views.

## 4. Discussion

This study investigated the neural mechanisms underlying the VWM-B program in children with DD. We performed a voxel-to-voxel intrinsic FC analysis to identify a network induced by VWM-B training. The results indicated that training with the VWM-B dual-task cognitive–motor program, compared to VWM single-task cognitive training, generated an intrinsic FC network encompassing the right and left postcentral gyrus (PostCG), left/right precuneus, left superior frontal gyrus (SFG), and left posterior division of supramarginal gyrus and angular gyrus (pSMG/AG). Both the right and left PostCG regions showed decreased FC, suggesting a reduction in motor task engagement due to a possible automation following VWM-B training. Conversely, increased FC in the bilateral precuneus, left SFG, and left pSMG/AG indicates a probable shift of cognitive resources towards the cognitive aspects of VWM-B. Each region in this network plays a crucial role in cognitive and motor functions related to DD, which is further discussed in the subsequent paragraphs.

In the estimated network, the results revealed a decrease in intrinsic FC in the right and left PostCG regions after training with VWM-B. The PostCG includes the primary somatosensory cortex and is a crucial brain region for proprioception, with a participation in the sensorimotor loop [39]. The literature indicates that individuals with DD face proprioceptive sensory impairments, with a strong correlation between proprioceptive acuity and reading abilities [40]. Children with DD struggle to integrate proprioceptive signals for balance control and attention, although their attentional control during stance can be significantly enhanced after receiving an intervention [41]. Proprioceptive interventions have been shown to improve reading skills, fluency, and written word recognition, underscoring better lexical access [42]. Proprioception-enhancing exercises have been demonstrated to be effective in improving reading performance in students with learning disorders [43]. Proprioception plays a crucial role in postural control [44]. Previous clinical trials have demonstrated that the VWM-B program positively affects postural control in children with DD [2,24]. Improved postural control was correlated with enhancements in working memory and reading skills [24]. The original study that the present study expanded on showed increased resting-state FC between the left fusiform gyrus and bilateral Crus II regions of the cerebellum, supporting the postural control and automaticity issues in dyslexia [2]. The increased FC of the Crus II regions probably stemmed from altered proprioceptive processing, as the cerebellar cortex is essential for this function [2,45]. It has been discussed that participants who received training with VWM-B showed higher performance in using non-visual information to maintain postural control and benefit from the visual information to perform cognitive functions [24]. They revealed that those participants benefited from vestibular and/or proprioceptive information and were less dependent on visual inputs to maintain postural control [24]. They also concluded that the motor strategies relating to balance control were automatized, and further neural resources were allocated to the cognitive task following the intervention by the VWM-B program [24]. Thus, the reduced intrinsic FC in the right and left PostCG regions may reflect the automatization of postural control in children with DD following VWM-B training. This program includes both passive and active states of balance tasks that utilize ankle and hip strategies [24]. Therefore, we conclude that engaging proprioceptive sensory input during these tasks helps automate both the balance-related movements and proprioceptive and sensorimotor processing, leading to a decrease in PostCG connectivity after fifteen sessions of VWM-B training.

It seems that after automating postural control motor strategies, neural resources were redirected to the cognitive task of the VWM-B program, resulting in increased intrinsic FC in other cognitive-related brain regions. The findings of the current study showed an increased intrinsic FC in the left/right precuneus, left SFG, and left pSMG/AG regions. The precuneus is the medial surface of the parietal cortex, located between the sensorimotor cortices of the paracentral lobule and the parieto-occipital cortex [46]. The precuneus is functionally linked to large-scale brain networks, including the dual default mode network and the para-cingulate network, a subnetwork of the central executive network [46]. These networks play a significant role in sensorimotor dynamics and working memory, with the precuneus primarily involved in visuospatial processing, visual integration, and memory retrieval [46,47]. The anterior precuneus is involved in sensorimotor processing, the central precuneus is involved in cognitive processing, and the posterior precuneus is involved in visuospatial processing [48]. The increased intrinsic FC of the left/right precuneus regions appears to result from certain aspects of the VWM-B program. This dual-task cognitive–motor training focuses on enhancing working memory through its cognitive task and improving postural control via its motor task, engaging both sensorimotor and visuospatial processing [2,24].

The left SFG is a crucial part of the neural network for working memory, activated during high-level executive processing, and it plays a role in spatial processing [49,50]. It implies that this region contributes to advanced working memory manipulation while maintaining a focus on spatial cognition. Certain subregions of the SFG correlated with both the cognitive control network and the cognitive execution network, while the posterior subregion of the SFG showed a correlation with sensorimotor-related brain regions [51]. It appears that mixing the visuospatial/sensorimotor balance task with the working memory maintenance subprocess in the VWM-B significantly impacted the intrinsic FC of the left SFG [24].

The current study aligns with evidence indicating the functional and structural connectivity between the PostCG and precuneus regions with the left supramarginal and angular gyri [52,53]. The role of these gyri within the inferior parietal lobule has been examined across various studies, showing correlations with reading-related cognitive functions such as semantic, phonological, and orthographic processing [54,55,56]. An earlier study highlighted their involvement in phonological recoding when visually reading words [57]. In Alzheimer’s patients, degeneration of the left supramarginal and angular gyri was linked to deficits in phonological and lexical skills, affecting writing abilities [58]. Individuals with lower orthographic competence struggled with automatic word recognition and exhibited reduced reading fluency, associated with decreased activation in these gyri [59]. Furthermore, the left supramarginal/angular gyri play a role in memory encoding [60], with research indicating that the posterior division of the supramarginal gyrus is primarily related to working memory. Its dorsal section shows significant activation in response to words, suggesting involvement in integrating sublexical and lexical cues [61]. It can be concluded that the left supramarginal/angular gyri are more activated during phonological and orthographic processing related to reading, which enhances word recognition and reading fluency. It is important to highlight the left pSMG’s specific role in working memory and lexical processing, as well as the AG’s specific function in reading [56,61]. Thus, the increased intrinsic FC of the left pSMG/AG following training with the VWM-B program is entirely justifiable given its characteristics. The previous studies confirmed the positive effects of VWM-B on working memory capacity and various reading skills [24,29,60]. VWM-B is the only dual-task cognitive–motor training program specifically designed to improve the reading skills of individuals with dyslexia, integrating reading practice with working memory and postural control tasks [24]. It can be concluded that this program could enhance the FC of the left pSMG/AG, leading to improved reading ability and working memory capacity in children with DD [2].

## 5. Limitations

Although the study’s results provided valuable insights into the neural mechanisms underlying the VWM-B program, there are some limitations. Head motion artifacts during fMRI data collection were the primary limitation of this study. Forcing a child to remain still or using multiple scans to obtain the desired data would be unethical [62]. While our study had a good sample size, a larger sample size could enhance statistical power and help in generalizing the results [2]. Although the difference in sex distribution between groups did not reach statistical significance (*p* = 0.055), the numerical imbalance should be considered a limitation and warrants conservative interpretation of the findings, particularly given the small sample size. The findings were based on short-term training with VWM-B; thus, the long-term effects of the program require further investigation, preferably using diffusion tensor imaging data [2]. The absence of concurrent behavioral measures may limit direct brain–behavior inference in the present study, so this work’s interpretations are supported by behavioral findings from the original investigation, which used the same dataset. Given that the program involves visuomotor training and includes certain visuospatial aspects, we recommend incorporating eye tracking data to assess its impact on eye movements and visual attention [24]. Beyond dyslexia, we also suggest examining VWM-B’s potential effects on neural plasticity in populations with ADHD, Parkinson’s disease, Alzheimer’s disease, and stroke, and among the elderly. Although the VWM-B robot is portable, its weight and difficulty in transport pose challenges; therefore, we recommend developing more accessible versions of VWM-B for neural research.

## 6. Conclusions

VWM-B is a cognitive–motor training program that positively impacts reading-related skills (phonological and orthographical processing), working memory, executive functions, and postural control in children with DD [2,24,29]. Previous studies using force plate CoP and fMRI data indicated that this program facilitated the automatization of postural control and redirected cognitive resources to cognitive functions [2,24]. This automatization correlated with improvements in reading ability and working memory [2,24]. The findings of the current study confirmed this automatization and the redirection of cognitive resources. The estimated network showed negative voxel-to-voxel intrinsic FC in the right and left PostCG regions, while it was positive in the left/right precuneus, left SFG, and left pSMG/AG regions. This suggests that the visuomotor and visuospatial aspects of VWM-B activated proprioception processing in the PostCG regions, which primarily process sensorimotor and proprioception tasks [39]. The negative intrinsic FC indicates reduced activation in these regions after fifteen training sessions, confirming the automatization of sensorimotor and proprioception processing. Consequently, more cognitive resources were redirected from the PostCG to cognitive regions, particularly the left/right precuneus and left SFG, enhancing their FC post-training. These regions work together in sensorimotor and visuospatial processing as well as cognitive functions related to working memory and reading [46,47,49,51]. Additionally, incorporating lexical content into the VWM-B working memory tasks increased FC in the left pSMG/AG, which is crucial for working memory and reading processes [56,61]. In conclusion, the study demonstrates that cognitive–motor dual-task training is more effective than single-task cognitive training in enhancing both cognitive and motor functions in children with DD. These findings emphasize the importance of postural control and automaticity in children with DD, supporting the automaticity theory in dyslexia [63,64].

## Figures and Tables

**Table 1:** Demographic characteristics.

Demography		Control (n = 8)	Experiment (n = 8)	Total (N = 16)	Group Differences (*p*-Value)
Mean (SD)					
Age (year)		8 (1.24)	8 (1.13)	8 (1.15)	u = 31.50 (0.955)
IQ level (WISC-IV)		92 (3.50)	95 (6.45)	93 (5.29)	t = 1.25 (0.175)
Attention level (CSI-4)		3 (1.19)	3 (1.98)	3 (1.58)	t = 0.153 (0.063)
Frequency (%)					
Gender	Boy	3 (37.50)	0 (0)	3 (18.70)	χ^2^ (1) = 3.69 (0.055)
	Girl	5 (62.50)	8 (100)	13 (81.30)	
School grade	First	4 (50.00)	3 (37.50)	7 (43.80)	χ^2^ (3) = 4.14 (0.246)
	Second	0 (0)	3 (37.50)	3 (18.80)	
	Third	3 (37.50)	1 (12.50)	4 (25.00)	
	Fourth	1 (12.50)	1 (12.50)	2 (12.40)	
Visual ability	Normal	8 (100)	7 (87.50)	15 (93.70)	χ^2^ (1) = 1.07 (0.302)
	Corrected	0 (0)	1 (12.50)	1 (6.30)	
Hearing ability	Normal	8 (100)	7 (87.50)	15 (93.70)	χ^2^ (1) = 1.07 (0.302)
	Corrected	0 (0)	1 (12.50)	1 (6.30)	
Disability	Reading	3 (37.50)	1 (12.50)	4 (25.00)	χ^2^ (2) = 2.33 (0.311)
	Reading/Writing	3 (37.50)	6 (75.00)	9 (56.30)	
	Reading/Writing/Math	2 (25.00)	1 (12.50)	3 (18.70)	

Abbreviations: WISC-IV, Wechsler intelligence scale for children—fourth edition. CSI-4, child symptoms inventory—fourth edition; total scores of 1 to 18 items of the parent checklist were considered. No significant differences were found in demographic data between the control and experiment groups.

**Table 2:** Voxel-to-voxel intrinsic functional connectivity network; mixed ANOVA outcomes.

Region	Clusters (x, y, z)	Voxel Size	Side	Connectivity	Beta (T = 14)	*p*-FDR	*p*-unc
PostCG	+52 −18 +54	74	right	negative	−2.13 (−5.55)	**0.000**	**0.000**
PostCG	−50 −26 +56	40	left	negative	−1.91 (−5.46)	**0.000**	**0.000**
Precuneous	−02 −66 +54	48	left/right	positive	1.54 (5.55)	**0.000**	**0.000**
SFG	−20 +26 +52	46	left	positive	1.43 (8.06)	**0.000**	**0.000**
pSMG/AG	−50 −50 +14	40	left	positive	1.27 (7.50)	**0.000**	**0.000**

Abbreviations: PostCG, postcentral gyrus; SFG, superior frontal gyrus; pSMG, posterior division of supramarginal gyrus; AG, angular gyrus; *p*-FDR, significance with correction for multiple comparisons using false discovery rate; *p*-unc, significance without correction for multiple comparisons. Note: Bolded values indicate statistically significant *p*-values (*p*-FDR corrected < 0.05 and *p*-uncorrected < 0.001).

## Data Availability

The data presented in this study are available upon request from the corresponding authors, in accordance with the informed consent agreement approved by the Ethics Committee. Individual participant data cannot be made publicly available.
